# Flow Diversion vs. Coiling for Large and Giant Intracranial Aneurysms: A Systematic Review and Meta-Analysis

**DOI:** 10.3390/jcm15041357

**Published:** 2026-02-09

**Authors:** Matteo Scalise, Leonardo Di Cosmo, Carlo Cossa, Nicolò Andreella, Camilla Micieli, Stefano Bendoni, Roberto Stefini, Delia Cannizzaro

**Affiliations:** 1 Department of Neurosurgery, Fondazione IRCCS Istituto Neurologico Carlo Besta, 20133 Milan, Italy; 2 Humanitas University, Via Rita Levi Montalcini 4, 20072 Pieve Emanuele, Italy; 3 Department of Translational Medicine, University of Ferrara, Via Luigi Borsari 46, 44121 Ferrara, Italy; 4 SC Radiologia e Diagnostica per Immagini, ASST Ovest Milanese, 20025 Legnano, Italy; 5 Radiotherapy and Radiosurgery Department, IRCCS Humanitas Research Hospital, 20089 Rozzano, Italy; 6 Department of Neurosurgery, ASST Ovest Milano Legnano Hospital, 20025 Legnano, Italy

**Keywords:** flow diversion, coiling, large intracranial aneurysms, giant intracranial aneurysms, intracranial aneurysms

## Abstract

**Background**: The management of large (≥10 mm) and giant (≥25 mm) intracranial aneurysms remains clinically challenging due to their elevated rupture risk, morbidity, and procedural complications, which pose a dilemma for both intervention and conservative management. Flow diversion (FD) has emerged as a promising endovascular approach, although its comparative safety and efficacy versus Coiling remain unclear. **Methods**: Following PRISMA guidelines, studies published between January 2000 and March 2025 were identified across PubMed, EMBASE, Scopus, and Web of Science. Outcomes assessed included aneurysm recurrence, complete occlusion, favorable clinical outcomes, procedure-related complications and mortality. Odds ratios (ORs) with 95% confidence intervals (CIs) were calculated, and heterogeneity and publication bias were assessed. **Results**: A total of 1893 patients (1256 FD, 637 Coiling) and 1915 aneurysms across 33 studies were included. FD significantly reduced recurrence compared to Coiling (8% vs. 27%; *p* = 0.0001) and showed a trend toward a higher rate of complete occlusion (*p* = 0.0571). However, FD had a modestly increased rate of hemorrhagic complications (*p* = 0.0495). No other significant differences were found in clinical outcomes, major complications, ischemic events, delayed rupture, or mortality. **Conclusions**: Both FD and Coiling are effective and generally safe for large and giant intracranial aneurysms. FD is associated with lower recurrence and a trend toward a higher rate of complete occlusion, with similar overall safety but slightly higher hemorrhagic risk. FD is emerging as a preferred first-line option for large and giant unruptured aneurysms, while coiling remains important for ruptured aneurysms or when anatomical constraints limit the use of FD.

## 1. Introduction

Intracranial aneurysms represent pathological dilatations of the cerebral vasculature. Recent meta-analytic evidence suggests that unruptured intracranial aneurysms occur in approximately 5.4% of the general adult population, with prevalence influenced by age, sex, and vascular risk factors such as hypertension and smoking [1]. While many remain asymptomatic, their potential to rupture and cause severe complications makes them a significant clinical concern.

Aneurysms are commonly classified according to size into small (<10 mm), large (≥10 mm), and giant (≥25 mm) lesions, a distinction that carries significant implications for both natural history and treatment strategy. Large and giant aneurysms constitute approximately 5% of all cases and are associated with higher rates of recurrence, neurological morbidity, and mortality compared to small aneurysms. Large aneurysm size and specific anatomical locations have been identified to be significant predictors of aneurysm rupture, underscoring the importance of size-based classification in clinical decision-making [2,3].

A larger size increases the likelihood of intraluminal thrombosis, mass effects on nearby structures, and rupture, with conservatively managed large and giant aneurysms presenting an annual rupture rate of 8–10% [4].

**Figure 1:**
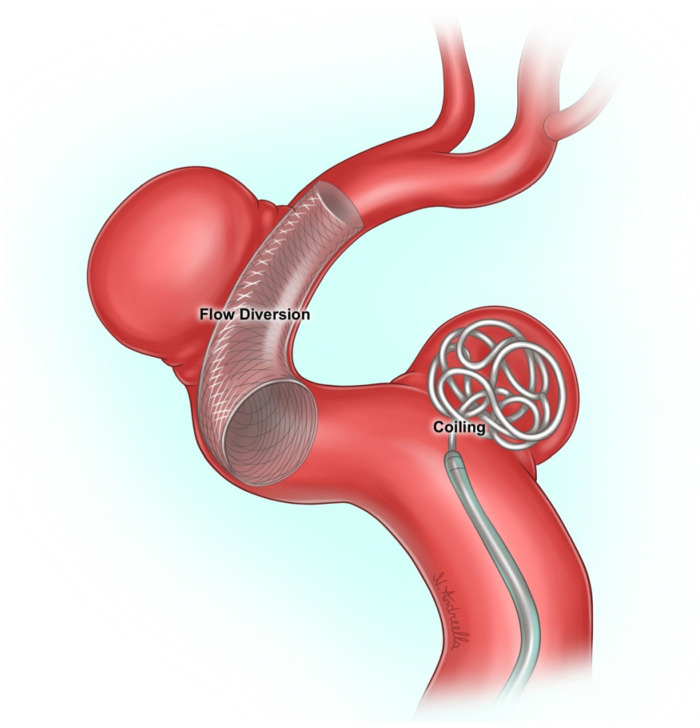
Schematic illustration comparing the mechanisms of FD and coiling in treating intracranial aneurysms. Disclosures: Figure 1 is an original illustration by Nicolò Andreella and was used with permission.

Management includes microsurgical clipping or endovascular interventions depending on aneurysm morphology, anatomical location, and patient-specific factors [5]. While microsurgical clipping continues to be widely utilized, endovascular techniques including coiling and flow diversion (FD) have gained increasing prominence, especially for aneurysms located in anatomically challenging areas or high-risk patients (Figure 1) [6,7].

In recent years, FD has emerged as a highly effective standalone therapy, showing superior occlusion rates coupled with comparatively low complication rates. FD devices are low-porosity stents deployed across the aneurysm neck to redirect blood flow away from the aneurysm, promoting gradual intra-aneurysmal thrombosis and progressive vessel wall remodeling. This is especially relevant in the context of these aneurysms as it does not cause the same significant hemodynamic changes frequently observed with coil embolization [8,9,10].

Endovascular coiling involves the placement of detachable coils within the aneurysm sac to induce thrombosis and exclude the aneurysm from the circulation. Coiling has previously been shown to be safe and effective when applied to large and giant aneurysms [10]. Although more often complicated by aneurysm recanalization, stent-assisted coiling has demonstrated lower rates of recurrence and hemorrhagic events [11].

Despite numerous studies, considerable debate remains regarding the optimal management of these aneurysms. While prior meta-analyses have evaluated FD and coiling across a wide range of aneurysm sizes and morphologies, few have specifically concentrated on large and giant aneurysms [12]. This meta-analysis addresses this gap by comparing the angiographic and clinical outcomes, and complication profiles associated with each treatment to inform evidence-based treatment decisions.

## 2. Materials and Methods

### 2.1. Literature Review Design

This systematic review was registered on PROSPERO (CRD420251052943) and conducted following PRISMA guidelines [13].

### 2.2. Search Strategy

We identified potential studies by searching four databases, PubMed/MEDLINE, Web of Science (WoS), Scopus, and EMBASE, from 1 January 2000, to 9 March 2025. The search strategy incorporated terms related to FD, coiling, and large or giant intracranial aneurysms; it was applied to titles, abstracts, and keywords. No language restrictions were imposed during the initial search. The full search strings are provided in the Supplementary Material (Table S1).

### 2.3. Eligibility Criteria

Four reviewers (M.S., C.C., N.A., L.D.C.) independently screened studies for eligibility and extracted relevant data. Any discrepancies were resolved through discussion with a senior author (D.C.).

Inclusion criteria were as follows: (1) studies involving exclusively adult patients (≥18 years) or providing outcomes stratified by adult versus pediatric populations; (2) studies enrolling patients with large (≥10 mm) and/or giant (≥25 mm) intracranial aneurysms; and (3) studies evaluating patients treated with FD (with or without adjunctive coiling) or coiling alone, either in single-arm designs or comparative cohorts, with outcomes clearly stratified by treatment modality.

Exclusion criteria were as follows: (1) studies involving exclusively pediatric populations (<18 years) or those that did not provide stratified outcomes for adult versus pediatric patients; (2) studies focusing solely on small aneurysms (<10 mm) or failing to report separate data for large (≥10 mm) and/or giant (≥25 mm) aneurysms; and (3) studies published in languages other than English.

Eligible designs included randomized controlled trials and prospective and retrospective cohort studies. Case reports, letters, conference abstracts, narrative reviews, and prior meta-analyses were excluded. Additionally, case series were excluded if they did not provide statistical data appropriate for inclusion in the quantitative synthesis.

### 2.4. Data Extraction

Data were extracted into a standardized spreadsheet. Variables included treatment modality; patient demographics (sample size, age, sex); clinical features (risk factors, aneurysm-related symptoms); aneurysm features (number, size category, mean diameter and neck size, rupture status, and location); follow-up details (mean duration and available cases); and angiographic and clinical outcomes (recurrence, morbidity, mortality, and treatment-related complications).

Data extraction was independently performed by three authors (C.C., L.D.C., N.A.) and subsequently reviewed and validated by a fourth author (M.S.).

### 2.5. Risk of Bias Assessment

The risk of bias of the included studies was independently assessed by two reviewers (L.D.C. and N.A.). Observational and non-randomized studies were evaluated using the ROBINS-I tool, while Randomized Controlled Trials (RCTs) were assessed using the Cochrane ROB-2 tool. Disagreements were resolved by a third reviewer (M.S.).

### 2.6. Statistical Analysis

Statistical analyses were conducted using the meta package in R (version 4.4.1). For each outcome, cumulative prevalence and corresponding 95% confidence intervals (CIs) were calculated and stratified by treatment modality. Prevalence estimates were logit-transformed and synthesized using a random-effects model to account for inter-study variability. Between-study heterogeneity was assessed using Higgins’ I^2^ statistic and visually represented via forest plots stratified by outcome and treatment group. To explore potential sources of heterogeneity and assess the influence of treatment modality on key outcomes, meta-regression analyses were performed. Publication bias was assessed with Egger’s test and funnel plots. Significance was set at *p* < 0.05.

## 3. Results

### 3.1. Study Selection

**Figure 2:**
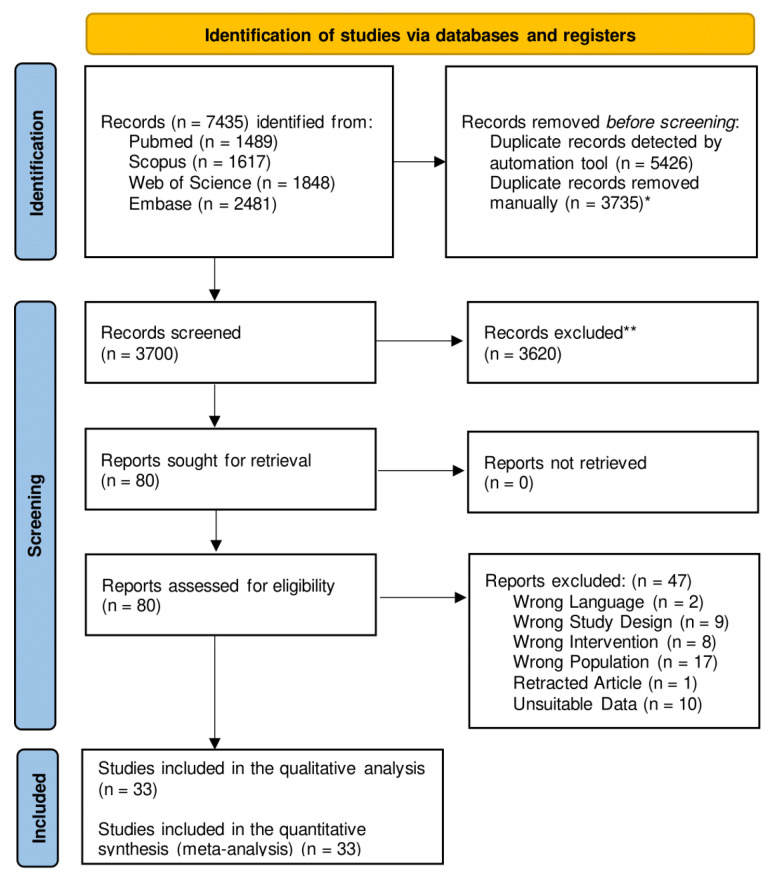
PRISMA flow chart breaking down the article screening, selection, and extraction process. * Deduplication done by M.S., C.C., N.A. Screening done by: M.S, C.C., N.A. ** All articles were excluded without the use of automation tools and all of them were manually screened by humans (M.S, C.C., N.A.) to avoid biases. Eventual conflicts have been solved.

The initial search retrieved 7435 articles. After the removal of 3735 duplicates, 3700 studies were screened based on their title and abstract. Of these, 3620 were excluded for not meeting the inclusion criteria. Full-text review of the remaining 80 articles identified 33 eligible studies for the final systematic review and meta-analysis (Table S2). The study selection process is illustrated in the PRISMA flow diagram in Figure 2.

### 3.2. Study and Patient Characteristics

Of the included studies, one was a randomized clinical trial, two were non-randomized clinical trials, and the remainder were retrospective cohort studies. Nine studies provided direct comparative data between patients treated with FD and those treated with coiling. Twenty studies exclusively reported outcomes in patients who underwent FD, while four focused solely on coiling.

Collectively, the studies comprised 1893 patients and 1915 aneurysms. Among these, 1256 patients underwent FD treatment in 29 studies, whereas 637 patients received coiling in 13 studies.

The pooled cohort included 1443 women (78.6%) and 392 men (21.4%), with a mean age of 57.95 years. Two studies (58 patients) did not report the sex distribution, and one study (7 patients) did not specify the mean age. Further study and patient characteristics are summarized in Table 1.

### 3.3. Aneurysm Characteristics

Among the analyzed aneurysms, 808 were classified as large and 302 as giant; in 805 cases (from 11 studies), the aneurysms were not stratified by size. The mean aneurysm diameter, calculated from 28 studies, was 19.9 mm; however, five studies did not report aneurysm size. Vessel location was specified for 1513 aneurysms (79%), with the most common one being the internal carotid artery (ICA) with 1210 cases (79.97%). Other locations included the vertebral artery (VA) in 105 cases (6.94%), the basilar artery (BA) in 62 cases (4.1%), and the vertebrobasilar junction (VBJ) in 21 cases (1.39%). Middle cerebral artery (MCA) aneurysms were identified in 34 cases (2.25%), while 59 aneurysms (3.9%) involved the posterior communicating artery (Pcomm). Less frequent locations included the anterior cerebral artery (ACA) in 9 cases (0.59%), the anterior communicating artery (Acomm) in 8 cases (0.53%), and the posterior cerebral artery (PCA) in 4 cases (0.26%). Only one aneurysm (0.07%) was located in the persistent primitive trigeminal artery. Of the total aneurysms, 1292 were unruptured and 92 were ruptured; notably, seven studies did not specify rupture status. Additional aneurysm characteristics are detailed in Table 2.

### 3.4. Risk of Bias

Of the 32 observational and non-randomized studies, most showed a moderate overall risk of bias, mainly due to confounding, participant selection, and outcome assessment (Figures S1 and S2). Three studies were identified as having a serious risk of bias, primarily related to confounding. The domain exhibiting the lowest risk across studies was deviations from intended interventions.

The sole included RCT was assessed as having a low risk of bias across all domains except for the randomization process, which raised some concerns (Figures S3 and S4).

Overall, although the evidence base offers important clinical insights, the methodological limitations identified necessitate cautious interpretation of the findings.

### 3.5. Angiographic Outcomes

Angiographic outcomes were primarily evaluated based on aneurysm occlusion rates utilizing the Raymond-Roy Occlusion Classification (RROC) for coiled aneurysms and the O’Kelly-Marotta (OKM) grading scale for FD. For analysis purposes, the results were stratified into complete occlusion (RROC Grade I or OKM Grade D) and partial occlusion (RROC Grades II–III or OKM Grades B–C).

**Figure 3:**
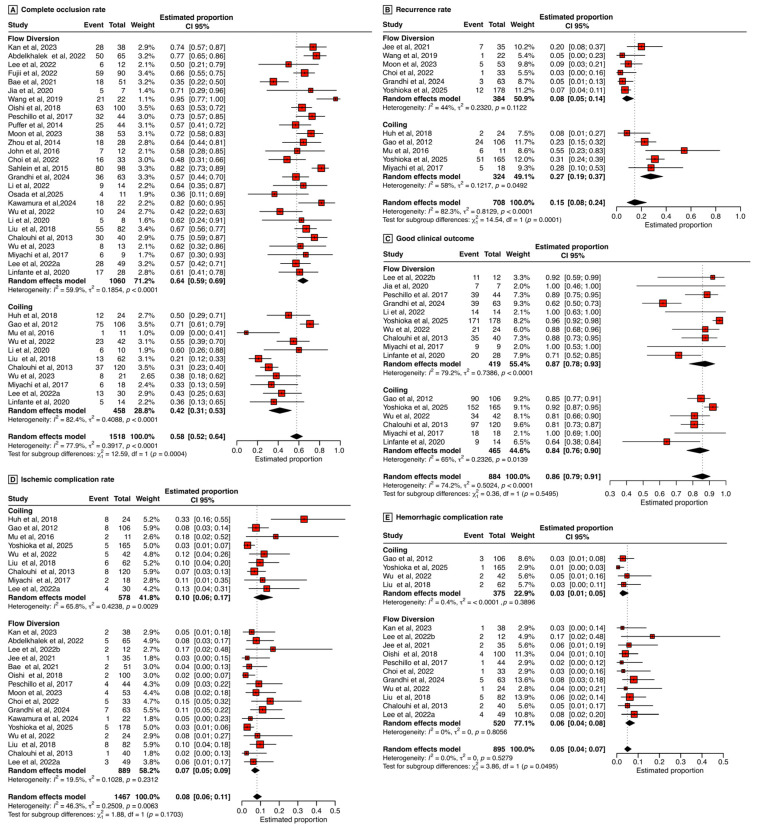
Forest plots summarizing key angiographic, clinical, and safety outcomes in patients treated with FD or coiling for large and giant intracranial aneurysms. (**A**) Complete occlusion rate. (**B**) Recurrence rate. (**C**) Good clinical outcome. (**D**) Ischemic complication rate. (**E**) Hemorrhagic complication rate [14,15,16,17,18,19,20,21,22,23,24,25,26,27,28,29,30,31,32,33,34,35,36,37,38,39,40,41,42,43,44,45,46].

A total of 1518 patients across 38 treatment arms were included in the analysis of complete occlusion rates. The mean angiographic follow-up duration was 16.46 months. The pooled OR favored FD with an OR of 1.78 (95% CI: 0.98–3.24), although this trend narrowly missed statistical significance (*p* = 0.0571). While the moderator analysis did not reach statistical significance, subgroup analysis showed a significant difference in occlusion rates between FD and coiling (χ^2^ = 12.59, *p* = 0.0004). Considerable heterogeneity was observed among studies (τ^2^ = 0.392, I^2^ = 77.9%, *p* < 0.0001), reflecting substantial variability in reported outcomes (Figure 3A).

For near-complete occlusion, data from 17 treatment arms encompassing 724 patients were analyzed, with a mean angiographic follow-up of 18.96 months. The pooled odds ratio suggested a trend toward higher partial occlusion rates with coiling (OR: 0.61; 95% CI: 0.32–1.20), although this did not reach statistical significance (*p* = 0.15). Moderate heterogeneity was observed among studies (τ^2^ = 0.347, I^2^ = 58.4%, *p* = 0.0006) (Figure S5A).

### 3.6. Clinical Outcomes

Twelve studies reported patient functional outcomes post-treatment, with favorable clinical outcomes defined as a modified Rankin Scale (mRS) score ≤ 2 at final follow-up. Pooled analyses demonstrated that both coiling and FD significantly improve clinical outcomes, with no statistically significant difference in efficacy between the two modalities (χ^2^ = 0.36, *p* = 0.5495) (Figure 3C). The model indicated significant residual heterogeneity among the included studies (τ^2^ = 0.502, I^2^ = 74.2%, *p* < 0.0001).

### 3.7. Procedure-Related Mortality

Procedure-related mortality was reported in 16 studies. Subgroup analysis revealed no significant difference in procedure-related mortality between FD and coiling (χ^2^ = 0.22, *p* = 0.6353), with a pooled odds ratio of 0.22 (95% CI: 0.077–0.636) for FD and 0.21 (95% CI: 0.085–0.52) for coiling. The heterogeneity of reported rates was moderate (τ^2^ = 0.444, I^2^ = 47.4%, *p* = 0.0119) (Figure S5B).

### 3.8. Recurrence Rate

Eleven studies comprising a total of 708 patients were included in the analysis of aneurysm recurrence rates, with 384 patients treated via FD and 324 via coiling. Recurrence rates were significantly lower in the FD cohort compared to the coiling group (*p* = 0.0001, Figure 3B). The pooled recurrence rate for FD was 8%, with moderate but non-significant heterogeneity observed among studies (τ^2^ = 0.232, I^2^ = 44.0%, *p* = 0.112). Conversely, the coiling group exhibited a substantially higher pooled recurrence rate of 27%, accompanied by significant heterogeneity across studies (τ^2^ = 0.122, I^2^ = 58%, *p* = 0.0492).

### 3.9. Delayed Rupture

Delayed rupture was defined as any aneurysmal rupture occurring post-treatment in cases where the aneurysm was initially deemed secured. The analysis included 10 studies with a markedly uneven distribution between treatment groups (FD = 8; Coiling = 2). The resultant heterogeneity was low (τ^2^ = 0.0818, I^2^ = 0.0%, *p* = 0.574) (Figure S5C). Moderator analysis revealed no significant impact of treatment modality on the risk of delayed rupture (*p* = 0.67), suggesting comparable outcomes between FD and coiling. The model explained none of the between-study variance (R^2^ = 0%).

Incidence rates were approximately 3% in the coiling cohort and 4% in the FD cohort, though the treatment group samples these values were derived from are limited.

### 3.10. Total Major Complications

Major complications were defined as hemorrhagic or ischemic events, including delayed aneurysm rupture. A total of 22 studies encompassing 27 treatment arms were included in the analysis. Pooled estimates revealed no significant difference in the incidence of major complications between FD and coiling. Residual heterogeneity was moderate (τ^2^ = 0.258, I^2^ = 56.2%, *p* = 0.0002) (Figure S5D).

Moderator analysis demonstrated that treatment modality did not significantly affect the risk of major complications (*p* = 0.59), with the model unable to account for the between-study variance (R^2^ = 0%).

### 3.11. Ischemic Complications

Ischemic complications were defined as any thromboembolic or ischemic events occurring peri- or post-procedurally, including transient ischemic attacks. The analysis encompassed 20 studies comprising 25 treatment arms. Residual heterogeneity was moderate (τ^2^ = 0.251, I^2^ = 46.3%, *p* < 0.0063). Pooled analysis revealed no significant effect of treatment modality on ischemic complication rates (*p* = 0.13) (Figure 3D).

### 3.12. Hemorrhagic Complications

Hemorrhagic complications were defined as any intracranial bleeding events occurring intra- or post-procedurally. Pooled analysis demonstrated a significant influence of treatment modality on hemorrhagic complication risk (*p* = 0.0495), indicating that FD was associated with a significantly increased risk compared to coiling (Figure 3E). The analysis included 13 studies encompassing 15 treatment arms. Interstudy heterogeneity was negligible (τ^2^ = 0, I^2^ = 0.0%, *p* = 0.528).

## 4. Discussion

The prognosis of large intracranial aneurysms is generally poor, and early treatment is typically advised. A Japanese study reported an annual rupture risk of 4.37% for aneurysms measuring between 10 and 24 mm, rising to 33.4% for those exceeding 24 mm [47]. Given the technical difficulty and potential for serious complications associated with surgical management, various endovascular approaches have been developed and are being increasingly favored as less invasive alternatives to conventional surgery. This study analyzed the two main endovascular techniques used in recent years for the treatment of large and giant aneurysms: coiling and FD. Our analysis directly compared the independent efficacy and safety of FD and coiling across 33 studies, including 1893 patients and 1915 large and giant IAs. Our findings suggest that while both modalities are effective in achieving favorable clinical outcomes, FD may confer advantages in certain critical domains.

### 4.1. Efficacy and Occlusion Outcomes

Angiographic outcomes remain fundamental to assessing the long-term efficacy of treating intracranial aneurysms. Coil embolization has shown lower initial occlusion rates (10–60%) and high recanalization rates (56–90%) in very large or giant aneurysms [48], whereas FD has achieved obliteration rates approaching 95% in regions such as the internal carotid artery [49].

Across 38 treatment arms and 1518 patients, FD showed a trend towards higher complete occlusion rates (OR 1.78; 95% CI: 0.98–3.24), narrowly missing statistical significance (*p* = 0.0571). Considerable heterogeneity was noted across studies (I^2^ = 82.61%), which was attributed to variability in aneurysm characteristics, device generations, procedural techniques, operator experience, and follow-up durations.

Regarding near-complete occlusion, coiling showed a non-significant trend for higher partial occlusion rates (OR 0.61; 95% CI: 0.32–1.20; *p* = 0.15).

These findings strengthen the evidence favoring FD for complex or large aneurysms but highlight the need for case-by-case treatment planning.

### 4.2. Clinical Outcomes and Mortality

Overall morbidity and mortality were comparable to previous reports. Procedure-related mortality was slightly lower for FD (5%) than coiling (7%), consistent with prior meta-analyses showing morbidity and mortality rates of 9.8% and 3.8% for FD [50]. Reported rates for coiling can reach up to 17% morbidity and 8% mortality for giant aneurysms [51].

Our results show that both FD and endovascular coiling demonstrate comparable rates of favorable functional outcomes and are generally safe in appropriately selected patient cohorts.

### 4.3. Aneurysm Recurrence

Analysis of recurrence rates revealed a superiority for FD over coiling. Across 11 studies comprising 708 patients, FD was associated with a significantly lower pooled recurrence rate of 8%, in contrast to 27% in the coiling cohort. These findings suggest greater FD durability, likely attributable to their capacity to promote endothelial remodeling and progressive aneurysm exclusion through vessel reconstruction rather than mere intraluminal packing [52].

The heterogeneity in the FD group did not reach statistical significance, suggesting reasonably consistent results across studies. In contrast, the coiling group exhibited substantial heterogeneity (I^2^ = 58%, *p* = 0.0492), likely reflect variability in coiling technique, operator experience, aneurysm morphology, and follow-up protocol employed.

This data aligns with prior literature indicating that in large and giant aneurysms, coiling alone is prone to higher rates of recurrence and retreatment, especially in lesions with wide necks or complex geometries [53,54]. In contrast, FD promotes hemodynamic changes and biologic healing that provide a more definitive effect, especially for complex anatomies less suited to coiling. Overall, these findings support using FD as a first-line option for selected high-risk aneurysms, particularly large unruptured cases, while underscoring the need for long-term imaging and tailored strategies for patients treated with coiling.

### 4.4. Complication Profile

Pooled analysis demonstrated no significant difference in major complication rates between FD and coiling.

The moderate residual heterogeneity reflects true variability due to factors like population, technique, and device generation, and the significant test for residual heterogeneity shows that the unexplained variability is likely influenced by operator experience and aneurysm morphology.

Moderator analysis confirmed that treatment type alone did not affect major complication risk (*p* = 0.59; R^2^ = 0%), reinforcing the importance of individualized treatment planning [55].

Ischemic complications, including thromboembolic events and transient ischemic attacks, were evaluated across 25 treatment arms and again showed no significant difference between FD and coiling (*p* = 0.13; I^2^ = 46.95%). This aligns with prior evidence suggesting ischemic risk highly depends on patient factors such as antiplatelet response and vascular anatomy [56,57].

Conversely, FD was associated with a significantly higher rate of hemorrhagic complications, including intra- and post-procedural intracranial hemorrhage, compared to coiling (*p* = 0.0495). Although the model did not explain between-study variance (R^2^ = 0%), this elevated risk may be attributable to factors inherent to FD, such as dual antiplatelet therapy requirements, vessel manipulation during device deployment, or delayed aneurysm thrombosis [58]. Further inquiry into risk stratification and peri-procedural management in patients undergoing FD is necessary.

Delayed rupture analysis revealed no significant difference in risk between modalities (*p* = 0.67). Although rare (~3–4%), this complication remains clinically devastating, warranting careful patient selection, particularly for FD cases where occlusion may be gradual [59].

### 4.5. Clinical and Research Implications

Taken together, our findings support the use of FD as a first-line treatment for large and giant unruptured intracranial aneurysms, particularly when long-term durability is a priority. Coiling, while still effective, may be more suitable in select scenarios—such as ruptured aneurysms, when rapid occlusion is critical, or when anatomical considerations limit the feasibility of FD.

From a policy and practice standpoint, these results emphasize the need for refined guidelines that stratify treatment strategies not only by aneurysm size and location but also by patient-specific risk profiles and the anticipated risk-benefit ratio of hemorrhagic complications.

### 4.6. Limitations

Several limitations merit acknowledgement. The predominance of retrospective and observational designs introduces potential confounding and selection biases. Heterogeneity in follow-up durations and outcome definitions may limit the comparability across studies. In addition, the broad time window covered by the available literature could introduce bias related to the progressive evolution of endovascular procedures over time, especially Flow Diverters. Analysis of delayed rupture and certain complications was constrained by underreporting and imbalanced group sizes. Some studies combined FD with adjunctive coiling but did not consistently report separate outcomes. As a result, the pooled outcomes presented reflect the overall performance of FD, irrespective of adjunctive techniques, thereby limiting the interpretability of the results. It is worth noting that comparing the outcomes of FD alone versus in combination with coiling was not an objective of this analysis and was therefore not specifically addressed. A major limitation is the lack of rupture-status stratification, as most included studies did not report outcomes separately for ruptured and unruptured aneurysms. This likely leads to underestimation of FD-related mortality and overestimation of coiling recurrence, reducing the accuracy of the comparative results. Additionally, differences in occlusion scales may affect interpretation. OKM D is less strict than RROC I, which could slightly overstate FD’s rate of complete occlusion. It should also be considered that as new FD devices continue to evolve, outcomes from earlier generations may underestimate current efficacy and safety. Furthermore, the increasing adoption of endosaccular devices in the treatment of large and giant aneurysms may warrant investigation in future studies.

### 4.7. Future Directions

Future randomized controlled trials are needed to validate these findings and explore specific patient and aneurysm characteristics that may predict better outcomes with one modality over the other. Additionally, longer-term follow-up data will be essential to more accurately assess recurrence and complication rates beyond the 12- to 24-month window that dominates the current literature.

## 5. Conclusions

This meta-analysis demonstrates that both FD and coiling are effective and generally safe for large and giant intracranial aneurysms. FD was associated with lower recurrence rates, likely due to its inherent vascular remodeling effect, despite immediate angiographic results comparable to coiling. Angiographic outcomes suggest a trend favoring FD for complete occlusion, although the considerable heterogeneity across studies underscores the need for individualized treatment decisions based on aneurysm characteristics and patient factors. Both modalities show comparable safety profiles regarding ischemic complications and overall morbidity and mortality. However, FD was associated with a moderately increased risk of hemorrhagic complications, likely related to its procedural complexity and antiplatelet requirements. Given these findings, FD emerges as a preferred first-line treatment for large and giant unruptured aneurysms, particularly where long-term durability is critical. Coiling remains important in select scenarios such as ruptured aneurysms or when anatomical constraints limit FD use. These results highlight the importance of tailored patient selection, careful peri-procedural management, and long-term follow-up. Future studies should aim to refine risk stratification, clarify sources of outcome variability, and support the development of consensus guidelines that consider aneurysm size, morphology, and patient-specific risk profiles.

## Figures and Tables

**Table 1:** Summary of patient characteristics in included studies.

Study	Procedure	Total Patients, N	Patients with Angiographic Follow-Up, N (%)	Mean Angiographic Follow-Up, Months ± SD
Yoshioka et al. 2025 [14]	FD	173	173 (100%)	16.7 ± 11.8
	Coiling	164	164 (100%)	32.0 ± 19.6
Wu et al. 2022 [15]	FD	24	18 (75%)	6.5
	Coiling	42	28 (66.7%)	10.5
Li et al. 2020 [16]	FD	8	8 (100%)	6.9 ± 4.0
	Coiling	10	5 (50%)	6.9 ± 4.0
Liu et al. 2018 [17]	FD	82	73 (89%)	6
	Coiling	62	53 (85.5%)	6
Chalouhi et al. 2013 [18]	FD	40	35 (87.5%)	7
	Coiling	120	90 (75%)	12
Wu et al. 2023 [19]	FD	13	9 (69.2%)	-
	Coiling	21	12 (57.1%)	-
Miyachi et al. 2017 [20]	FD	9	9 (100%)	6
	Coiling	18	18 (100%)	6
Lee. et al. 2022a [21]	FD	49	45 (91.8%)	30.3 ± 18.7
	Coiling	30	28 (93.3%)	40.8 ± 25.6
Linfante et al. 2020 [22]	FD	28	22 (78.6%)	-
	Coiling	14	10 (71.4%)	-
Kan et al. 2023 [23]	FD	38	36 (94.7%)	12
Abdelkhalek et al. 2022 [24]	FD	65	60 (92.3%)	12 ± 8.6
Lee et al. 2022b [25]	FD	12	10 (83.3%)	6
Fujii et al. 2022 [26]	FD	84	71 (84.5%)	36
Jee et al. 2021 [27]	FD	35	-	-
Bae et al. 2021 [28]	FD	51	51 (100%)	19.1
Jia et al. 2020 [29]	FD	7	7 (100%)	57.5 ± 16.7
Wang et al. 2019 [30]	FD	22	21 (95.5%)	12.2 ± 0.7
Oishi et al. 2018 [31]	FD	94	86 (91.4%)	10.2 ± 5.6
Peschillo et al. 2017 [32]	FD	44	41 (93.1%)	20.7
Puffer et al. 2014 [33]	FD	44	36 (81.8%)	10.9
Moon et al. 2023 [34]	FD	53	53 (100%)	12
Zhou et al. 2014 [35]	FD	28	25 (89.3%)	9.9
John et al. 2016 [36]	FD	12	10 (83.3%)	18.2
Choi et al. 2022 [37]	FD	33	33 (100%)	9.4 ± 6.8
Sahlein et al. 2015 [38]	FD	98	98 (100%)	6
Grandhi et al. 2024 [39]	FD	63	50 (79.4%)	9.1 ± 6.5
Li et al. 2022 [40]	FD	14	12 (85.7%)	18
Osada et al. 2025 [41]	FD	11	11 (100%)	12
Kawamura et al. 2024 [42]	FD	22	22 (100%)	29.6
Huh et al. 2018 [43]	Coiling	24	16 (66.7%)	27.2
Gao et al. 2012 [44]	Coiling	102	79 (77.5%)	38.1
Mu et al. 2016 [45]	Coiling	11	9 (81.8%)	28.7
Kim et al. 2000 [46]	Coiling	19	15 (78.9%)	11.9

**Table 2:** Summary of aneurysm characteristics in patients in included studies.

Study	Procedure	Total Aneurysms, N	Large (≥10 mm)	Giant (≥25 mm)	Mean Size, mm	Unruptured, N (%)	Ruptured, N (%)
Yoshioka et al. 2025 [14]	FD	178	139 (78.1%)	39 (21.9%)	22.0	-	-
	Coiling	165	155 (93.9%)	10 (6.10%)	15.8	-	-
Wu et al. 2022 [15]	FD	24	24 (100%)	0 (0.00%)	13.3	24 (100%)	0 (0.00%)
	Coiling	42	42 (100%)	0 (0.00%)	13.2	30 (71.4%)	12 (28.6%)
Li et al. 2020 [16]	FD	8	0 (0.00%)	8 (100%)	26.9	8 (100%)	0 (0.00%)
	Coiling	10	0 (0.00%)	10 (100%)	31.3	10 (100%)	0 (0.00%)
Liu et al. 2018 [17]	FD	82	-	-	18.0	82 (100%)	0 (0.00%)
	Coiling	62	-	-	17.1	62 (100%)	0 (0.00%)
Chalouhi et al. 2013 [18]	FD	40	-	-	14.9	40 (100%)	0 (0.00%)
	Coiling	120	-	-	14.9	120 (100%)	0 (0.00%)
Wu et al. 2023 [19]	FD	-	-	-	-	-	-
	Coiling	-	-	-	-	-	-
Miyachi et al. 2017 [20]	FD	9	8 (88.9%)	1 (11.1%)	16.6	9 (100%)	0 (0.00%)
	Coiling	18	17 (94.4%)	1 (6.60%)	14.6	18 (100%)	0 (0.00%)
Lee et al. 2022a [21]	FD	49	37 (75.5%)	12 (24.5%)	22.0	49 (100%)	0 (0.00%)
	Coiling	30	29 (96.7%)	1 (3.30%)	18.3	30 (100%)	0 (0.00%)
Linfante et al. 2020 [22]	FD	-	-	-	-	-	-
	Coiling	-	-	-	-	-	-
Kan et al. 2023 [23]	FD	38	38 (100%)	0 (0.00%)	12.2	-	-
Abdelkhalek et al. 2022 [24]	FD	65	50 (76.9%)	15 (23.1%)	16.4	65 (100%)	0 (0.00%)
Lee et al. 2022b [25]	FD	12	12 (100%)	0 (0.00%)	15.9	12 (100%)	0 (0.00%)
Fujii et al. 2022 [26]	FD	90	-	-	16.6	90 (100%)	0 (0.00%)
Jee et al. 2021 [27]	FD	35	24 (68.6%)	11 (31.4%)	18.3	35 (100%)	0 (0.00%)
Bae et al. 2021 [28]	FD	51	-	-	21.9	51 (100%)	0 (0.00%)
Jia et al. 2020 [29]	FD	7	7 (100%)	0 (0.00%)	-	7 (100%)	0 (0.00%)
Wang et al. 2019 [30]	FD	22	11 (50.0%)	11 (50.0%)	24.5	22 (100%)	0 (0.00%)
Oishi et al. 2018 [31]	FD	100	-	-	16.9	100 (100%)	0 (0.00%)
Peschillo et al. 2017 [32]	FD	44	37 (84.1%)	7 (15.9%)	-	40 (90.9%)	4 (9.10%)
Puffer et al. 2014 [33]	FD	44	23 (52.3%)	21 (47.7%)	20.9	-	-
Moon et al. 2023 [34]	FD	53	-	-	19.6	53 (100%)	0 (0.00%)
Zhou et al. 2014 [35]	FD	28	20 (71.4%)	8 (26.6%)	21.6	28 (100%)	0 (0.00%)
John et al. 2016 [36]	FD	12	6 (50.0%)	6 (50.0%)	27.6	12 (100%)	0 (0.00%)
Choi et al. 2022 [37]	FD	33	6 (18.2%)	27 (81.8%)	19.5	33 (100%)	0 (0.00%)
Sahlein et al. 2015 [38]	FD	98	-	-	14.7	98 (100%)	0 (0.00%)
Grandhi et al. 2024 [39]	FD	63	0 (0.00%)	63 (100%)	29.0	61 (96.8%)	2 (3.20%)
Li et al. 2022 [40]	FD	14	14 (100%)	0 (0.00%)	17.0	14 (100%)	0 (0.00%)
Osada et al. 2025 [41]	FD	11	9 (81.8%)	2 (18.2%)	20.4	11 (100%)	0 (0.00%)
Kawamura et al. 2024 [42]	FD	22	-	-	18.4	22 (100%)	0 (0.00%)
Huh et al. 2018 [43]	Coiling	24	11 (45.8%)	13 (54.2%)	26	15 (62.5%)	9 (37.5%)
Gao et al. 2012 [44]	Coiling	106	75 (70.8%)	31 (29.2%)	17.2	41 (38.7%)	65 (61.3%)
Mu et al. 2016 [45]	Coiling	11	-	-	-	-	-
Kim et al. 2000 [46]	Coiling	19	14 (73.7%)	5 (26.3%)	18.4	-	-

## Data Availability

All extracted data are available from the references listed in Table S2. Data used for all analyses, analytic code and any other materials used in the review are available upon request.

## Supplementary Materials

The following supporting information can be downloaded at: https://www.mdpi.com/article/10.3390/jcm15041357/s1, PRISMA 2020 checklist; PRISMA 2020 abstract checklist; Figure S1: Traffic light summary plot of cohort studies evaluated through the ROBINS-I; Figure S2: Summary plots of ROBINS-I; Figure S3: Traffic light summary plot of RCTs evaluated through the ROB2; Figure S4: Summary plot of ROB2; Figure S5: Forest plots summarizing key angiographic, clinical, and safety outcomes in patients treated with flow diversion or coiling for large and giant intracranial aneurysms. (A) Near Complete occlusion rate. (B) Procedure related mortality. (C) Delayed Rupture. (D) Major complications.; Table S1: Search Strings used for Database literature search; Table S2: Summary list of all included studies.

## Abbreviations

The following abbreviations are used in this manuscript:

ACA	Anterior Cerebral Artery
Acomm	Anterior Communicating Artery
BA	Basilar Artery
CI	Confidence Interval
FD	Flow Diversion
IAs	Intracranial Aneurysms
ICA	Internal Carotid Artery
MCA	Middle Cerebral Artery
mRS	Modified Rankin Scale
OKM	O’Kelly-Marotta grading scale
OR	Odds ratio
PCA	Posterior Cerebral Artery
Pcom	Posterior Communicating Artery
PRISMA	Preferred Reporting Items for Systematic Reviews and Meta-Analyses
RCT	Randomized Controlled Trial
RoB	Risk of Bias
ROBINS	Risk of Bias in Non-randomized Studies of Interventions
RROC	Raymond-Roy Occlusion Classification
SE	Standard Error
VA	Vertebral Artery
VBJ	Vertebrobasilar Junction
WoS	Web of Science
