# Supplementary material for: Flow Diversion vs. Coiling for Large and Giant Intracranial Aneurysms: A Systematic Review and Meta-Analysis
Source: J Clin Med. 2026 Feb 9;15(4):1357. doi: 10.3390/jcm15041357 (PMC12942218; doi:10.3390/jcm15041357)
Supplement: Supplementary file 1 [file jcm-15-01357-s001.zip › jcm-4094174-supplementary.pdf]

Supplementary material

Flow Diversion versus Coiling for Large and Giant Intracranial Aneurysms: a Systematic Review and Meta-Analysis

Figure S1: Traffic light summary plot of cohort studies evaluated through the ROBINS-I. 2
Figure S2: Summary plots of ROBINS-I 3
Figure S3: Traffic light summary plot of RCTs evaluated through the ROB2. 4
Figure S4: Summary plot of ROB2 5
Figure S5: Forest plots summarizing key angiographic, clinical, and safety outcomes in patients treated with flow diversion or coiling for large and giant intracranial aneurysms. (A) Near Complete occlusion rate. (B) Procedure related mortality. (C) Delayed Rupture. (D) Major complications. 6
Table S1: Search Strings used for Database literature search 7
Table S2: Summary list of all included studies. 10

Figure S1: Traffic light summary plot of cohort studies evaluated through the ROBINS-I.

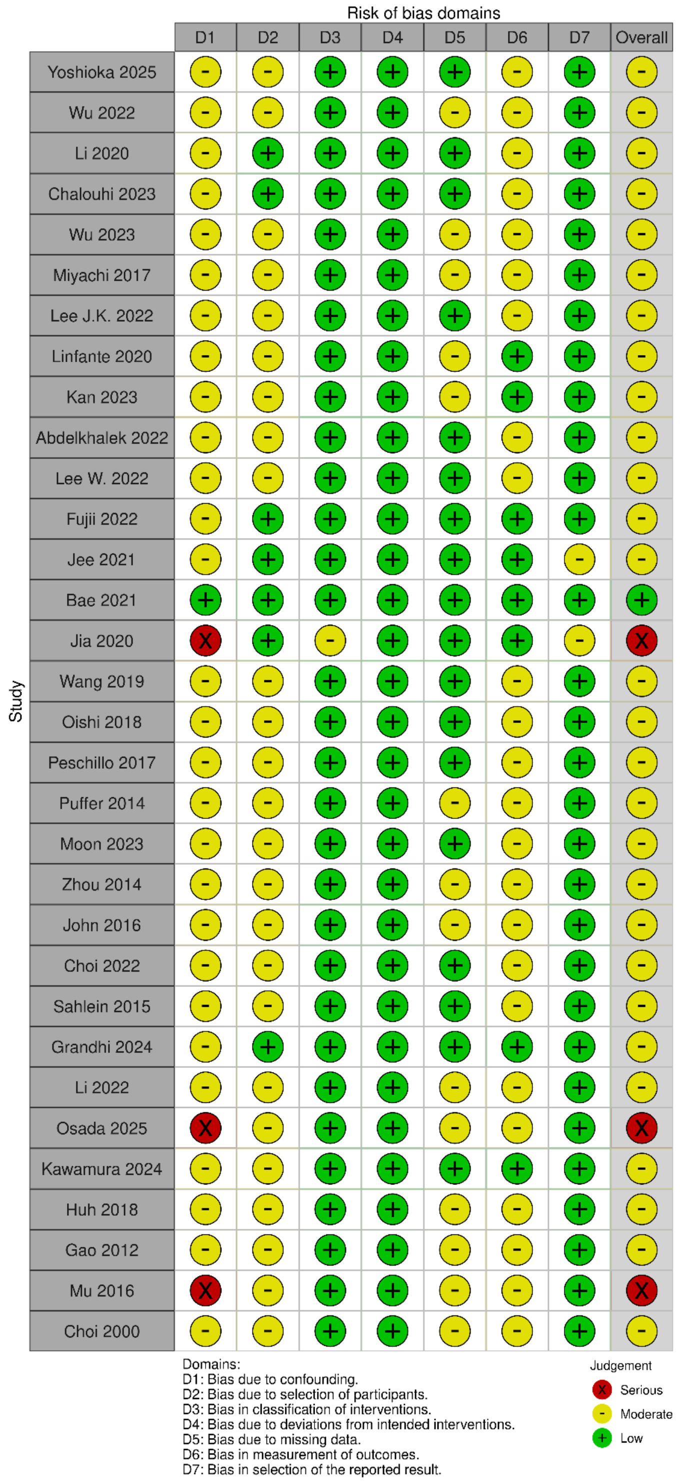

Figure S2: Summary plots of ROBINS-I

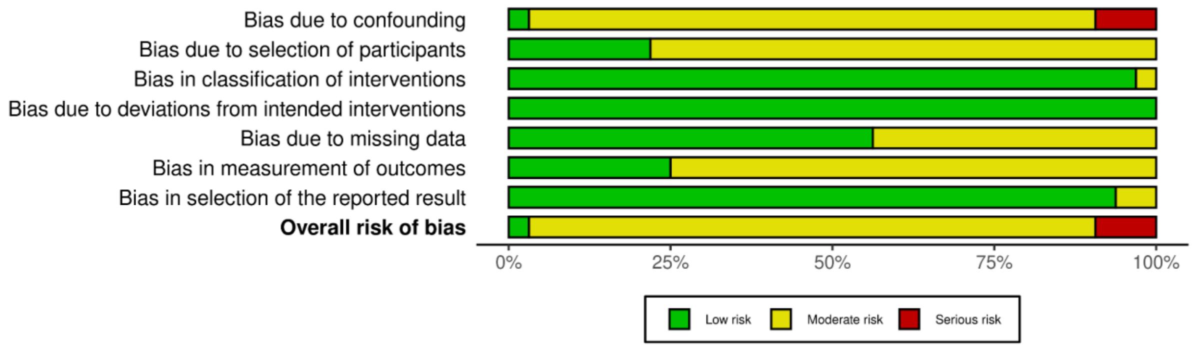

Figure S3: Traffic light summary plot of RCTs evaluated through the ROB2.

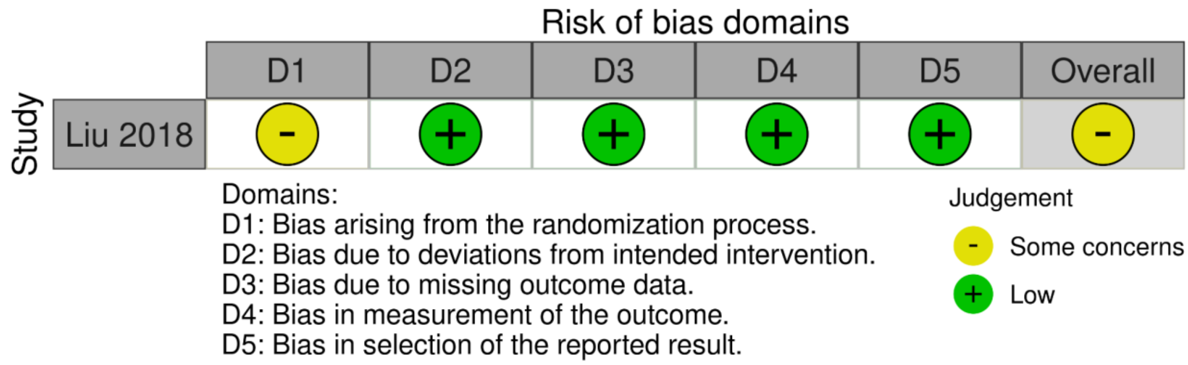

Figure S4: Summary plot of ROB2

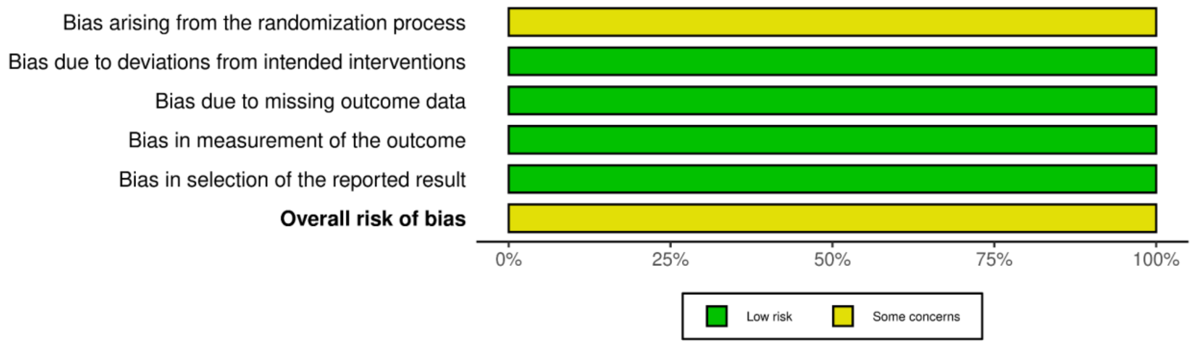

Figure S5: Forest plots summarizing key angiographic, clinical, and safety outcomes in patients treated with flow diversion or coiling for large and giant intracranial aneurysms. (A) Near Complete occlusion rate. (B) Procedure related mortality. (C) Delayed Rupture. (D) Major complications.

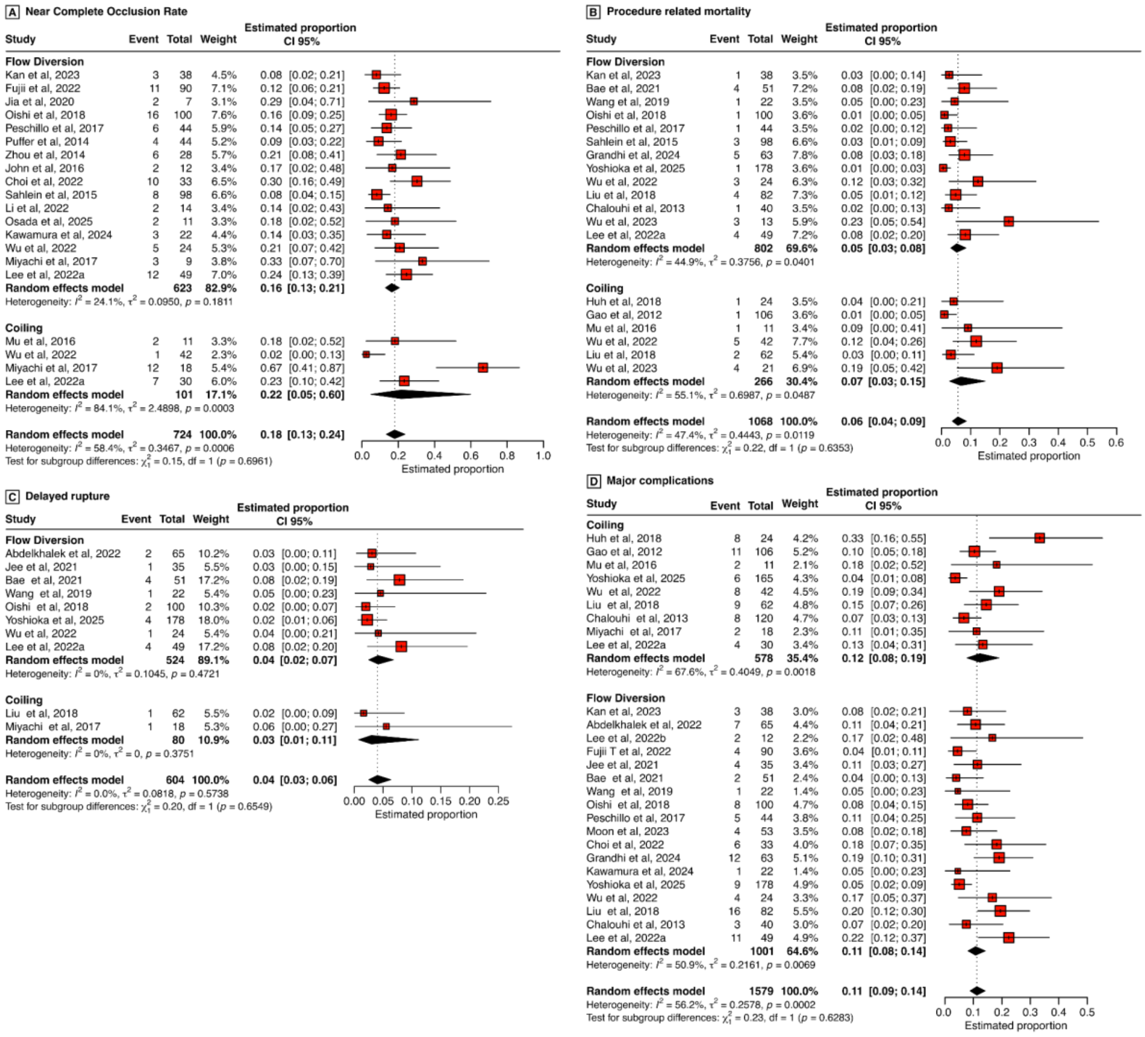

Table S1: Search Strings used for Database literature search

Pubmed	Scopus	Web of Science	Embase
(("Flow Diverter" OR "Flow Diverters" OR "Endovascular Flow Diverter" OR "Endovascular Flow Diverters" OR "Pipeline" OR "Flow Diversion") AND ("Coiling" OR "Endovascular Coiling" OR "Endovascular Embolization" OR "Stent-Assisted Coiling")) OR ("Endosaccular" OR "Endosaccular Device" OR "Endosaccular Devices" OR "Endosaccular Flow Diversion" OR "Endosaccular Flow Diverter" OR "Intrasaccular" OR	(TITLE-ABS-KEY("Flow Diverter" OR "Flow Diverters" OR "Endovascular Flow Diverter" OR "Endovascular Flow Diversers" OR "Pipeline" OR "Flow Diversion") AND TITLE-ABS-KEY("Coiling" OR "Endovascular Coiling" OR "Endovascular Embolization" OR "Stent-Assisted Coiling")) OR TITLE-ABS-KEY("Endosaccular" OR "Endosaccular Device" OR "Endosaccular Devices" OR "Endosaccular Flow Diversion" OR "Endosaccular Flow	(TS=("Flow Diverter" OR "Flow Diverters" OR "Endovascular Flow Diverter" OR "Endovascular Flow Diversers" OR "Pipeline" OR "Flow Diversion") AND TS=("Coiling" OR "Endovascular Coiling" OR "Endovascular Embolization" OR "Stent-Assisted Coiling")) OR TS=("Endosaccular" OR "Endosaccular Device" OR "Endosaccular Devices" OR "Endosaccular Flow Diversion" OR "Endosaccular Flow Diverter" OR "Intrasaccular" OR	("Flow Diverter"/exp OR "Flow Diverters"/exp OR "Endovascular Flow Diverter"/exp OR "Endovascular Flow Diversers"/exp OR "Pipeline"/exp OR "Flow Diversion"/exp) AND ("Coiling"/exp OR "Endovascular Coiling"/exp OR "Endovascular Embolization"/exp OR "Stent-Assisted Coiling"/exp) OR ("Endosaccular"/exp OR "Endosaccular Device"/exp OR "Endosaccular Devices"/exp OR "Endosaccular Flow Diversion"/exp OR "Endosaccular Flow Diverter"/exp OR

"Intrasaccular Device" OR "Intrasaccular Devices" OR "Intrasaccular Flow Diversion" OR "Intrasaccular Flow Diverter" OR "Woven EndoBridge" OR "WEB") AND ("Large Intracranial Aneurysm" OR "Large Intracranial Aneurysms" OR "Giant Intracranial Aneurysm" OR "Giant Intracranial Aneurysms" OR "Large Aneurysm" OR "Large Aneurysms" OR "Giant Aneurysm" OR "Giant Aneurysms" OR "Intracranial Aneurysm" OR "Intracranial Aneurysms" OR "Cerebral Aneurysm" OR "Cerebral Aneurysms" OR	Diverter" OR "Intrasaccular" OR "Intrasaccular Device" OR "Intrasaccular Devices" OR "Intrasaccular Flow Diversion" OR "Intrasaccular Flow Diverter" OR "Woven EndoBridge" OR "WEB") AND TITLE-ABS-KEY("Large Intracranial Aneurysm" OR "Large Intracranial Aneurysms" OR "Giant Intracranial Aneurysm" OR "Giant Intracranial Aneurysms" OR "Large Aneurysm" OR "Large Aneurysms" OR "Giant Aneurysm" OR "Giant Aneurysms" OR "Intracranial Aneurysm" OR "Intracranial Aneurysms" OR "Cerebral Aneurysm" OR "Cerebral	"Intrasaccular Device" OR "Intrasaccular Devices" OR "Intrasaccular Flow Diversion" OR "Intrasaccular Flow Diverter" OR "Woven EndoBridge" OR "WEB") AND TS=("Large Intracranial Aneurysm" OR "Large Intracranial Aneurysms" OR "Giant Intracranial Aneurysm" OR "Giant Intracranial Aneurysms" OR "Large Aneurysm" OR "Large Aneurysms" OR "Giant Aneurysm" OR "Giant Aneurysms" OR "Intracranial Aneurysm" OR "Intracranial Aneurysms" OR "Cerebral Aneurysm" OR "Cerebral	"Intrasaccular"/exp OR "Intrasaccular Device"/exp OR "Intrasaccular Devices"/exp OR "Intrasaccular Flow Diversion"/exp OR "Intrasaccular Flow Diverter"/exp OR "Woven EndoBridge"/exp OR "WEB"/exp) AND ("Large Intracranial Aneurysm"/exp OR "Large Intracranial Aneurysms"/exp OR "Giant Intracranial Aneurysm"/exp OR "Giant Intracranial Aneurysms"/exp OR "Large Aneurysm"/exp OR "Large Aneurysms"/exp OR "Giant Aneurysm"/exp OR "Giant Aneurysms"/exp OR "Intracranial Aneurysm"/exp OR "Intracranial Aneurysms"/exp OR "Cerebral Aneurysm"/exp OR "Cerebral Aneurysms"/exp OR

"Aneurysm Treatment")	Aneurysms" OR "Aneurysm Treatment")	Aneurysms" OR "Aneurysm Treatment")	"Aneurysm Treatment"/exp)

Table S2: Summary list of all included studies.

**Study, Year**	**Study Name**
**Yoshioka et al. 2025**	Treatment of Unruptured Large and Giant Carotid Cavernous Aneurysms in Japan at the Time of Flow Diverter Introduction: A Nationwide, Multicenter Survey by the Japanese Society on Surgery for Cerebral Stroke
**Wu et al. 2022**	Flow Diversion vs. Stent-Assisted Coiling in the Treatment of Intradural Large Vertebrobasilar Artery Aneurysms
**Li et al. 2020**	Unfavorable Outcomes Related to Endovascular Treatment of Giant Vertebrobasilar Aneurysms
**Liu et al. 2018**	Parent artery reconstruction for large or giant cerebral aneurysms using the tubridge flow diverter: A multicenter, randomized, controlled clinical trial (PARAT)
**Chalouhi et al. 2013**	Comparison of flow diversion and coiling in large unruptured intracranial saccular aneurysms
**Wu et al. 2023**	Complications after endovascular treatment of large basilar trunk aneurysms
**Miyachi et al. 2017**	Innovations in Endovascular Treatment Strategies for Large Carotid Cavernous Aneurysms-The Safety and Efficacy of a Flow Diverter
**Lee. et al. 2022a**	Recovery from Cranial Nerve Symptoms after Flow Diversion without Coiling for Unruptured Very Large and Giant ICA Aneurysms
**Linfante et al. 2020**	Endovascular Treatment of Giant Intracranial Aneurysms
**Kan et al. 2023**	Treatment of large and giant posterior communicating artery aneurysms with the Surpass streamline flow diverter: Results from the SCENT trial
**Abdelkhalek et al. 2022**	Predictors of flow diverter stent in large and giant unruptured intracranial aneurysms, single-center experience
**Lee et al. 2022b**	Flow diverter for the treatment of large (> 10 mm) vertebral artery dissecting aneurysms
**Fujii et al. 2022**	Long-term Follow-up Results after Flow Diverter Therapy Using the Pipeline Embolization Device for Large or Giant Unruptured Internal Carotid Artery Aneurysms: Single-center Retrospective Analysis in the Japanese Population
**Jee et al. 2021**	Treatment Outcomes After Single-Device Flow Diversion for Large or Giant Aneurysms
**Bae et al. 2021**	Predictors of the effects of flow diversion in very large and giant aneurysms
**Jia et al. 2020**	Evaluating the Tubridge™ flow diverter for large cavernous carotid artery aneurysms
**Wang et al. 2019**	Variation of Mass Effect After Using a Flow Diverter With Adjunctive Coil Embolization for Symptomatic Unruptured Large and Giant Intracranial Aneurysms
**Oishi et al. 2018**	Flow diverter therapy using a pipeline embolization device for 100 unruptured large and giant internal carotid artery aneurysms in a single center in a Japanese

	population
**Peschillo et al. 2017**	Endovascular treatment of large and giant carotid aneurysms with flow-diverter stents alone or in combination with coils: A multicenter experience and long-term follow-up
**Puffer et al. 2014**	Treatment of cavernous sinus aneurysms with flow diversion: Results in 44 patients
**Moon et al. 2023**	Mass Effect After Flow Diversion for Unruptured Large and Giant Cavernous or Paraclinoid Internal Carotid Artery Aneurysm
**Zhou et al. 2014**	A Novel Flow-Diverting Device (Tubridge) for the Treatment of 28 Large or Giant Intracranial Aneurysms: A Single-Center Experience
**John et al. 2016**	Long-Term Effect of Flow Diversion on Large and Giant Aneurysms: MRI-DSA Clinical Correlation Study
**Choi et al. 2022**	A Single Flow Re-direction Endoluminal Device for the Treatment of Large and Giant Anterior Circulation Intracranial Aneurysms
**Sahlein et al. 2015**	Neuroophthalmological outcomes associated with use of the Pipeline Embolization Device: analysis of the PUFS trial results
**Grandhi et al. 2024**	Treatment of giant intracranial aneurysms using the Pipeline flow-diverting stent: Long-term results from the International Retrospective Study of the Pipeline Embolization Device (IntrePED) study
**Li et al. 2022**	Pipeline flex embolization device for the treatment of large unruptured posterior circulation aneurysms: Single-center experience
**Osada et al. 2025**	Intraoperative aneurysm flow analysis predicts intracranial large and giant aneurysm occlusion after flow diversion
**Kawamura et al. 2024**	Second-stage pipeline embolization device treatment with coil embolization for large cerebral aneurysm prevents silent diffusion-weighted image ischemic infarction: a retrospective study
**Huh et al. 2018**	Endosaccular treatment of very large and giant intracranial aneurysms with parent artery preservation: Single center experience with long term follow-up
**Gao et al. 2012**	A single-centre experience and follow-up of patients with endovascular coiling of large and giant intracranial aneurysms with parent artery preservation
**Mu et al. 2016**	Reconstructive Endovascular Treatment of Spontaneous Symptomatic Large or Giant Vertebrobasilar Dissecting Aneurysms: Clinical and Angiographic Outcomes
**Kim et al. 2000**	GDC embolisation of cavernous internal carotid artery aneurysms with parent artery preservation
